# Mandibular Radiation Dose Modifies the Association Between Post-Chemoradiotherapy Dental Extraction Timing and Osteoradionecrosis Risk: A Retrospective Cohort Study

**DOI:** 10.3390/cancers18111756

**Published:** 2026-05-27

**Authors:** Erkan Topkan, Efsun Somay, Sibel Bascil, Duriye Ozturk, Ugur Selek

**Affiliations:** 1 Department of Radiation Oncology, Faculty of Medicine, Başkent University, Adana 06790, Turkey; docdretopkan@gmail.com; 2 Department of Oral and Maxillofacial Surgery, Faculty of Dentistry, Başkent University, Ankara 06490, Turkey; 3 Department of Periodontology, Faculty of Dentistry, Başkent University, Ankara 06490, Turkey; bascil5@yahoo.com; 4 Department of Radiation Oncology, School of Medicine, Afyonkarahisar Health Sciences University, Afyonkarahisar 03340, Turkey; duriyeozturk07@gmail.com; 5 Department of Radiation Oncology, School of Medicine, Koç University, Istanbul 34010, Turkey; ugurselek@yahoo.com

**Keywords:** nasopharyngeal neoplasms, osteoradionecrosis, tooth extraction, radiotherapy dosage, mandible, chemoradiotherapy

## Abstract

**Background/Objectives**: This retrospective study evaluated whether the association between post-CCRT dental extraction timing and osteoradionecrosis of the jaw (ORNJ) risk is modified by mandibular radiation exposure in patients with locally advanced nasopharyngeal carcinoma (LA-NPC) treated with definitive concurrent chemoradiotherapy (CCRT). **Methods**: A total of 247 patients who did not undergo pre-CCRT dental extraction but underwent post-CCRT extraction were analyzed. Mandibular radiation exposure was quantified using EQD2. Associations between clinical, tumoral, dental, and dosimetric variables and ORNJ were assessed using multivariable logistic regression, including assessment of EQD2–timing interaction. ROC analyses were performed to evaluate discriminative performance and estimate exploratory thresholds. ORNJ developed in 23 patients (9.3%). Mandibular EQD2 was independently associated with ORNJ risk (OR 2.10 per 5 Gy; 95% CI 1.40–3.15; *p* < 0.001). A significant interaction between EQD2 and extraction timing was observed (OR 1.07; 95% CI 1.03–1.11; *p* = 0.023). EQD2 demonstrated excellent discrimination (AUC 0.885; cutoff 46.5 Gy), whereas extraction timing showed modest discrimination (AUC 0.691; cutoff 10 months). At <46.5 Gy, ORNJ rates remained low regardless of timing (1.0% vs. 3.9%). At ≥46.5 Gy, delayed extraction was associated with substantially higher ORNJ incidence (31.1% vs. 11.3%). Number of extracted teeth (OR 1.16; *p* = 0.012) and hemoglobin level (OR 0.62; *p* = 0.007) were also independent predictors. **Conclusions**: The effect of post-CCRT dental extraction timing on ORNJ risk is modified by mandibular radiation dose, supporting interpretation in conjunction with mandibular radiation exposure rather than as an isolated factor.

## Simple Summary

Osteoradionecrosis of the jaw (ORNJ) is a serious complication that may occur after radiotherapy or chemoradiotherapy for nasopharyngeal cancer, particularly following tooth extraction. It remains unclear whether the timing of tooth extraction after treatment independently affects this risk. In this study, we examined patients who underwent tooth extraction only after completing chemoradiotherapy and evaluated the combined effect of extraction timing and the radiation dose received by the jawbone. We observed that radiation dose was the main factor associated with risk. At lower dose levels, ORNJ was uncommon regardless of the timing of the tooth extraction. In contrast, at higher dose levels, later extraction was associated with a higher ORNJ risk. These results suggest that the timing of tooth extraction after chemoradiotherapy should not be considered in isolation but rather in relation to radiation exposure to support more individualized decision-making in clinical practice.

## 1. Introduction

Concurrent chemoradiotherapy (CCRT) represents the cornerstone of curative treatment for patients with locally advanced nasopharyngeal carcinoma (LA-NPC). The introduction of intensity-modulated radiotherapy (IMRT) has substantially improved locoregional tumor control while reducing several radiation-related toxicities compared with conventional radiotherapy techniques [1,2,3]. Consequently, the population of long-term survivors of NPC continues to increase, shifting greater clinical attention toward late treatment-related complications affecting craniofacial structures.

Among these complications, osteoradionecrosis of the jaw (ORNJ) remains one of the most serious late toxicities of head and neck radiotherapy. Clinically, ORNJ is characterized by exposed, non-healing irradiated bone that persists for several months and may lead to chronic pain, infection, fistula formation, and pathologic fracture, ultimately resulting in significant impairment of oral function and quality of life [4,5,6]. Notably, ORNJ has been reported in up to 10% of patients undergoing radiotherapy for head and neck cancer, even in the era of intensity-modulated radiotherapy (IMRT) and advanced modalities such as proton therapy, underscoring its persistence as a clinically significant and debilitating late toxicity. Despite advances in radiotherapy planning and delivery, ORNJ continues to occur in a subset of patients treated for head and neck malignancies.

The pathogenesis of ORNJ is complex and multifactorial. Radiation exposure induces a cascade of biological alterations within the mandibular microenvironment, including endothelial injury, microvascular compromise, hypocellularity, tissue hypoxia, and progressive fibroatrophic remodeling [7,8,9]. These changes impair the capacity for normal tissue repair and create a chronically compromised bone environment in which relatively minor traumatic events may precipitate tissue breakdown and necrosis.

In this biologically compromised setting, the magnitude of radiation dose delivered to the mandible represents an important contributor to ORNJ susceptibility. Several studies have demonstrated strong associations between mandibular dose–volume parameters and the development of ORNJ, suggesting that radiation exposure establishes a baseline level of bone vulnerability to subsequent injury [10,11,12]. Within this framework, ORNJ development may be conceptualized as a two-step process in which radiation exposure may contribute to a biologically vulnerable bone environment, while subsequent traumatic events act as triggering insults that precipitate tissue breakdown.

Within this context, traumatic insults to irradiated mandibular bone—particularly dental extraction—are widely recognized as major precipitating factors for ORNJ development. Consequently, comprehensive dental assessment and the prophylactic removal of non-restorable teeth prior to radiotherapy have become standard components of multidisciplinary head and neck cancer care [13,14,15]. Nevertheless, a substantial proportion of patients require dental extraction after completion of radiotherapy due to progressive radiation-associated dental deterioration, periodontal disease, or structural tooth damage. Although post-CCRT dental extraction is widely recognized as a major risk factor for ORNJ, the temporal relationship between extraction timing and ORNJ development remains insufficiently defined. Clinical practice varies considerably, with some clinicians advocating delayed extraction in the belief that longer healing intervals after radiotherapy may reduce ORNJ risk, whereas others suggest that progressive fibrosis and vascular compromise may increase susceptibility to trauma-induced necrosis over time. Evidence addressing this issue remains limited and heterogeneous [16]. Whether the risk associated with post-CCRT dental extraction is primarily determined by its timing or by the underlying level of mandibular radiation exposure remains unclear.

Importantly, the coexistence of pre- and post-CCRT dental extractions in many previous studies may confound the interpretation of the specific impact of post-CCRT dental trauma on ORNJ risk. Dental extraction performed before CCRT may independently alter mandibular vascularity, tissue integrity, and healing capacity, thereby obscuring the isolated contribution of post-CCRT extractions to ORNJ development [15,16]. By separating these two clinical scenarios, the temporal influence of post-CCRT dental trauma can be evaluated more accurately.

Therefore, the present study focused exclusively on patients with LA-NPC treated with definitive CCRT who did not undergo dental extraction before CCRT but subsequently underwent dental extraction after treatment completion. Within this carefully defined cohort, we investigated the association between the timing of post-CCRT dental extraction and the development of mandibular ORNJ while accounting for the radiation dose delivered to the mandible, specifically to determine whether mandibular radiation exposure modifies the effect of extraction timing on ORNJ risk.

## 2. Patients and Methods

### 2.1. Study Design and Ethical Approval

This retrospective cohort study was approved by the Institutional Review Board (IRB) of Başkent University Medical Faculty (project no: D-KA-2058; approval date: June 2022). The study was conducted in accordance with the Declaration of Helsinki and its subsequent amendments. All patients had provided written informed consent at the time of treatment, including consent for the use of their anonymized clinical, pathological, and treatment-related data for scientific research and publication, as part of routine clinical practice at our institution. This study was reported in accordance with the Strengthening the Reporting of Observational Studies in Epidemiology (STROBE) guidelines [17].

### 2.2. Patients

Patients were identified through a systematic review of institutional records of consecutively treated individuals with LA-NPC who received definitive CCRT at the Department of Radiation Oncology, Başkent University Medical Faculty, between June 2010 and December 2021.

Eligible patients were 18–80 years of age, had an Eastern Cooperative Oncology Group (ECOG) performance status of 0–1, and had histologically confirmed nonkeratinizing or undifferentiated nasopharyngeal carcinoma. All patients received at least one cycle of chemotherapy during radiotherapy. Clinical staging was determined according to the American Joint Committee on Cancer (AJCC) 8th edition staging system, including patients with T1–4N1–3M0 or T3–4N0–3M0 disease. All patients underwent pretreatment evaluation, including head and neck magnetic resonance imaging (MRI), 18F-fluorodeoxyglucose positron emission tomography/computed tomography (FDG PET-CT), a detailed ear–nose–throat examination, and a documented pretreatment dental assessment as part of routine multidisciplinary management. Baseline complete blood count and serum biochemistry parameters, including hemoglobin levels, were obtained from routine laboratory assessments performed within 7 days prior to initiation of CCRT. Clinical variables, including ECOG performance status, tobacco use, alcohol consumption, diabetes status, and laboratory parameters, were extracted from prospectively maintained institutional medical records.

To isolate the specific impact of post-CCRT dental trauma on ORNJ risk, patients who underwent dental extraction prior to initiation of CCRT were excluded. Additional exclusion criteria included prior head and neck radiotherapy, induction chemotherapy before CCRT, synchronous malignancies, immunosuppressive disorders, or insufficient follow-up documentation to determine whether post-CCRT dental extraction or ORNJ occurred.

**Figure 1:**
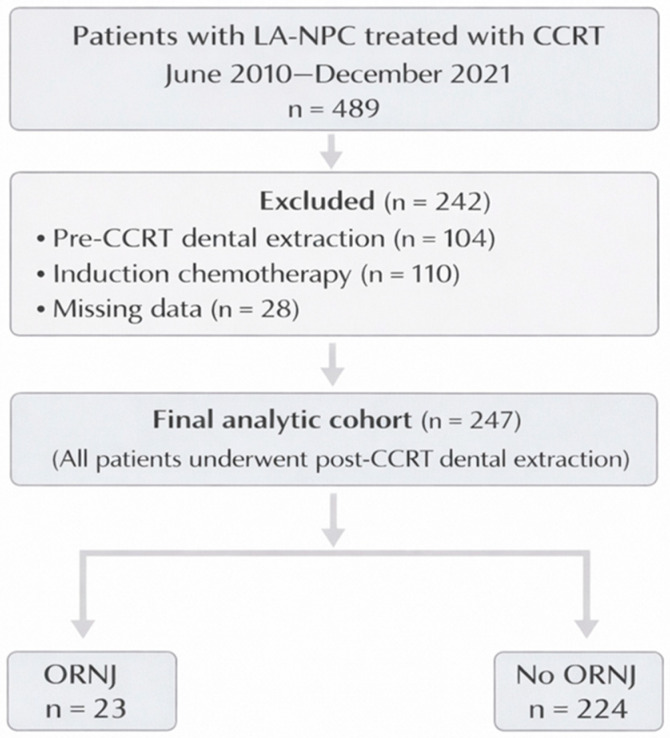
Patient selection and derivation of the final analytic cohort. **Note:** Among 489 patients with locally advanced nasopharyngeal carcinoma treated with concurrent chemoradiotherapy (CCRT) between June 2010 and December 2021, 242 were excluded (pre-radiotherapy dental extraction, n = 104; induction chemotherapy, n = 110; missing data, n = 28). The final analytic cohort comprised 247 patients who underwent post-CCRT dental extraction, including 23 who developed osteoradionecrosis of the jaw (ORNJ) and 224 who did not. **Abbreviations:** LA-NPC, locally advanced nasopharyngeal carcinoma; CCRT, concurrent chemoradiotherapy; ORNJ, osteoradionecrosis of the jaw.

Radiotherapy treatment planning records enabling the extraction of mandibular dose–volume parameters were required for inclusion. After application of these criteria, the final analytic cohort consisted exclusively of patients who did not undergo dental extraction before CCRT but subsequently underwent dental extraction after its completion, enabling evaluation of the temporal relationship between post-CCRT dental extraction timing and the development of mandibular ORNJ. The patient selection process and derivation of the final analytic cohort are illustrated in Figure 1.

### 2.3. Chemoradiotherapy Protocol

All patients received definitive CCRT in accordance with institutional treatment protocols for locally advanced nasopharyngeal carcinoma. Radiotherapy was delivered using simultaneous integrated boost intensity-modulated radiotherapy (SIB-IMRT) as previously described [18]. Treatment was delivered once daily, five days per week. The prescribed radiation doses were 70 Gy to the primary tumor and involved lymph nodes, 59.4 Gy to high-risk subclinical disease, and 54 Gy to elective nodal regions, delivered in 33 fractions.

Concurrent chemotherapy consisted of cisplatin administered every three weeks at a dose of 75–80 mg/m^2^ during radiotherapy. All patients received at least one cycle of concurrent platinum-based chemotherapy. Patients were advised to receive two additional cycles of adjuvant cisplatin-based doublet chemotherapy.

Supportive care measures were provided throughout treatment and included pain and mucositis management, antiemetic therapy, intravenous hydration when indicated, and nutritional support to maintain adequate caloric intake.

### 2.4. Pretreatment Oral Examination and Dental Management

All potentially eligible patients underwent a comprehensive dental evaluation prior to CCRT initiation. The assessment was performed by an experienced oral and maxillofacial surgeon (ES) and included detailed clinical oral examination and panoramic radiography, consistent with established recommendations for dental management in patients undergoing head and neck radiotherapy [19,20].

Dental caries were evaluated using illuminated dental mirrors and explorers in accordance with World Health Organization diagnostic criteria [21]. Periodontal status, tooth mobility, oral hygiene condition, and the presence of periapical pathology were clinically assessed as part of routine pretreatment dental evaluation. Teeth demonstrating extensive carious destruction not amenable to restorative treatment, inadequate periodontal support, advanced mobility, or significant periapical pathology unsuitable for endodontic therapy were classified as non-restorable. When indicated, such teeth were extracted at least 10 days prior to the initiation of CCRT. Teeth with superficial or moderate carious lesions were managed with appropriate restorative procedures. In addition, all patients received oral hygiene instruction and dental prophylaxis prior to treatment initiation. Patients who underwent dental extraction prior to initiation of CCRT were subsequently excluded from the present analytic cohort, as illustrated in Figure 1.

### 2.5. Post-CCRT Dental Extraction and Follow-Up

Upon completion of CCRT, patients were systematically monitored in accordance with the institutional follow-up protocol for NPC. Patients underwent clinical evaluations every 3 months during the first 2 years after CCRT, every 6 months between years 3 and 5, and annually thereafter, or more frequently if clinically indicated. At each follow-up visit, comprehensive oral examinations were performed to evaluate dental status and identify potential complications related to irradiation of the oral cavity and mandible.

Post-CCRT dental extractions were performed when teeth exhibited irreversible structural or periodontal compromise, including extensive dental caries not amenable to restoration, advanced periodontal disease with severe mobility, non-restorable crown destruction, persistent periapical infection, or symptomatic retained root fragments. Teeth considered clearly non-restorable or associated with advanced uncontrolled infection at pretreatment assessment were preferentially extracted before initiation of CCRT according to institutional dental management protocols. Teeth undergoing post-CCRT extraction had initially been considered clinically preservable at baseline evaluation but subsequently deteriorated during follow-up because of progressive radiation-associated dental deterioration and periodontal complications. Whenever possible, conservative dental management was preferred; however, extraction was performed when preservation of the affected tooth was deemed clinically unfeasible.

For each patient undergoing dental extraction following CCRT, the timing of extraction was defined as the interval between completion of CCRT and the date of the extraction procedure. When multiple extractions occurred, the earliest post-CCRT extraction was used for timing analyses.

### 2.6. ORNJ Evaluation

Patients were systematically evaluated for ORNJ during routine follow-up examinations. ORNJ was defined as the presence of exposed irradiated mandibular bone or radiologic evidence of mandibular necrosis persisting for at least three months in the absence of tumor recurrence or metastasis, in accordance with widely accepted diagnostic criteria [6,7].

Diagnosis was primarily established through clinical examination, including persistent bone exposure, mucosal disruption, or fistula formation within previously irradiated mandibular regions. When clinically indicated, radiologic imaging modalities, including panoramic radiography, computed tomography, or MRI, were used to confirm the diagnosis and assess the extent of bony involvement. All suspected cases were reviewed by a multidisciplinary team comprising radiation oncologists and oral and maxillofacial surgeons to confirm the diagnosis and exclude tumor recurrence.

ORNJ severity was classified according to the Notani staging system [22]. However, for the purposes of the present study, ORNJ was analyzed as a binary outcome, defined as the development of clinically confirmed ORNJ at any time during follow-up.

### 2.7. Mandibular Contouring and Dosimetric Analysis

Mandibular radiation dose parameters were extracted from the original radiotherapy treatment planning data for all eligible patients. The mandible was contoured as an organ at risk (OAR) on planning computed tomography scans according to established head and neck radiotherapy contouring guidelines [23]. Contouring was performed using bone window settings to facilitate accurate delineation of cortical and trabecular bone structures.

The mandibular contour encompassed the entire osseous mandible, including the alveolar processes, symphysis, body, angles, rami, coronoid processes, and bilateral mandibular condyles. Teeth were contoured as a separate structure per institutional protocol and were therefore excluded from the mandibular volume. Adjacent soft tissues and temporomandibular joint structures were not included. Dose–volume histograms (DVHs) generated from the treatment planning system were reviewed to obtain mandibular dosimetric parameters.

The mean mandibular dose (Dmean) was selected as the primary dosimetric variable, as it reflects overall radiation exposure of mandibular bone and has been widely reported as a clinically relevant predictor of ORNJ risk, despite the focal nature of radiation-induced injury at extraction sites [15,16,17,18].

Mandibular radiation doses were converted to equivalent dose in 2 Gy fractions (EQD2) using the linear–quadratic model with an α/β ratio of 3 Gy, consistent with late-responding normal tissues, to account for spatial variation in total dose and fraction size within the mandible. EQD2 was calculated using the following formula:

EQD2 =D×(d + α/β)/(2 + α/β)

where D represents the total delivered dose and d denotes the dose per fraction.

### 2.8. Statistical Analysis

The primary endpoint was the development of ORNJ during follow-up. The study aimed to evaluate whether mandibular radiation dose modifies the association between the timing of post-CCRT dental extraction and ORNJ risk. Statistical analyses were performed using SPSS version 26 (IBM Corp., Armonk, NY, USA) and R (R Foundation for Statistical Computing, Vienna, Austria). Continuous variables were summarized as medians with ranges and categorical variables as frequencies and percentages. Analyses were performed using a complete-case approach because patients with insufficient clinical, extraction-related, ORNJ, or dosimetric documentation were excluded during cohort selection. Continuous variables were compared between patients with and without ORNJ using either the Student’s *t* test or the Mann–Whitney U test according to data distribution, whereas categorical variables were analyzed using the χ^2^ test or Fisher’s exact test, as appropriate. Receiver operating characteristic (ROC) analyses were subsequently applied to evaluate the discriminative capacity of continuous variables associated with ORNJ and to estimate exploratory cutoff values for mandibular EQD2, post-CCRT extraction timing, number of extracted teeth, and hemoglobin level. Thresholds were estimated using the maximum Youden index; however, continuous variables were retained in their original form in regression analyses to avoid information loss, and ROC-derived thresholds were used only for descriptive comparisons.

Associations between candidate variables and ORNJ were first evaluated using univariable logistic regression, and variables with *p* < 0.05 were subsequently included in multivariable logistic regression models, given the binary nature of the endpoint. Because mandibular mean dose and EQD2 represent closely related dosimetric parameters, only EQD2 was included to avoid multicollinearity. Continuous predictors were modeled as continuous variables, and the effect of mandibular EQD2 was expressed as a per-5 Gy increase to improve clinical interpretability. Given that the primary objective was to evaluate effect modification rather than time-to-event outcomes, logistic regression was selected as the appropriate analytical framework. Effect modification was assessed by including an interaction term between mandibular EQD2 and the interval from CCRT completion to dental extraction, enabling a more nuanced characterization of dose–timing interplay. Statistical significance of the interaction was evaluated using likelihood ratio tests comparing nested models. Multicollinearity among predictors was assessed using variance inflation factors (*VIF*s). Model calibration was evaluated using the Hosmer–Lemeshow goodness-of-fit test, and internal validity was assessed through bootstrap resampling with 1000 iterations. Restricted cubic spline modeling was used to explore potential nonlinear relationships between mandibular radiation dose and ORNJ probability across extraction timing intervals, and joint dose–timing effects were visualized using interaction plots and risk heatmaps. All statistical tests were two-sided, and *p* values < 0.05 were considered statistically significant, with analyses reported in accordance with the STROBE Statement.

## 3. Results

### 3.1. Patient Cohort and Follow-Up

A total of 489 patients with locally advanced nasopharyngeal carcinoma treated with definitive CCRT between June 2010 and December 2021 were identified from institutional records. After application of the predefined eligibility criteria, 247 patients remained and constituted the final analytic cohort, all of whom underwent post-CCRT dental extraction.

The median follow-up duration for the analytic cohort was 74 months (range, 2–158 months). During follow-up, ORNJ developed in 23 of the 247 patients (9.3%).

### 3.2. Baseline and Treatment Characteristics

Baseline clinical, tumor, lifestyle, dental, and dosimetric characteristics are presented in Table 1. The median age of the analytic cohort was 56 years (range, 18–78 years), and 163 patients (66.0%) were male. Demographic characteristics, histologic subtype, tumor stage, nodal stage, tobacco use, and alcohol consumption were comparable between patients with and without ORNJ.

In contrast, several dental and treatment-related variables differed significantly between groups. Patients who developed ORNJ had lower baseline hemoglobin levels (median 10.46 vs. 12.56 g/dL; *p* < 0.001), a greater number of extracted teeth (median 5 vs. 3; *p* = 0.004), and a longer interval between completion of CCRT and dental extraction (median 10 vs. 8 months; *p* = 0.010). Mandibular radiation exposure also differed significantly, with higher mean mandibular dose (median 56.4 Gy vs. 32.8 Gy; *p* < 0.001) and EQD2 (median 53.1 Gy vs. 26.2 Gy; *p* < 0.001) observed in patients who developed ORNJ.

### 3.3. Exploratory ROC Analysis

Exploratory ROC curve analyses were conducted to assess the discriminative performance of candidate predictors for ORNJ (Table 2). Mandibular EQD2 demonstrated excellent discriminative ability, with an area under the curve (AUC) of 0.885 (95% CI 0.831–0.939). The optimal cutoff value estimated using the maximum Youden index was 46.5 Gy, corresponding to a sensitivity of 91.7% and a specificity of 89.9%. This threshold was used to describe ORNJ incidence across EQD2 categories.

Extraction timing after completion of CCRT showed modest discriminative performance (AUC 0.691; 95% CI 0.573–0.799; sensitivity: 67.5%; specificity: 67.1%), whereas the number of extracted teeth (AUC 0.801; 95% CI 0.696–0.906; sensitivity: 75.2%; specificity: 72.3%) and baseline hemoglobin level (AUC 0.807; 95% CI 0.691–0.912; sensitivity: 73.9%; specificity: 72.3%) demonstrated moderate discriminative ability. The corresponding exploratory cutoff values were 10 months for extraction timing, 3 extracted teeth, and 11.4 g/dL for hemoglobin.

To facilitate descriptive interpretation, ORNJ incidence was compared across these ROC-derived thresholds (Table 3). ORNJ occurred in 20.4% of patients with mandibular EQD2 ≥46.5 Gy compared with 2.0% among those with lower doses (*p* < 0.001). Similarly, ORNJ developed in 24.1% of patients whose dental extraction was performed ≥10 months after CCRT, compared with 5.2% among those undergoing extraction earlier (*p* < 0.001). ORNJ incidence was also higher among patients with ≥3 extracted teeth compared with those with fewer extractions (19.0% vs. 7.3%; *p* = 0.027), and among patients with hemoglobin ≤11.4 g/dL compared with those with higher levels (21.3% vs. 2.5%; *p* < 0.001).

These ROC-derived thresholds were used solely for descriptive comparisons. To avoid information loss associated with dichotomization, all variables were retained in their original continuous form in subsequent regression analyses.

### 3.4. Univariable Logistic Regression Analysis

Associations between candidate clinical, dental, and dosimetric variables and the development of osteoradionecrosis of the jaw (ORNJ) were evaluated using univariable logistic regression analyses (Table 4). Mandibular radiation exposure demonstrated the strongest association with ORNJ risk. Mandibular EQD2 was significantly associated with ORNJ development, with each 5 Gy increase corresponding to an approximately twofold increase in odds (OR 1.93; 95% CI 1.53–2.44; *p* < 0.001).

Additional variables significantly associated with ORNJ included the number of extracted teeth (OR 1.23 per additional tooth; 95% CI 1.07–1.41; *p* = 0.004), extraction timing after CCRT (OR 1.07 per month; 95% CI 1.02–1.12; *p* = 0.009), and hemoglobin level (OR 0.63 per g/dL increase; 95% CI 0.48–0.82; *p* < 0.001). In contrast, age, sex, tobacco use, alcohol consumption, histologic subtype, and nodal stage were not significantly associated with ORNJ.

### 3.5. Multivariable Analysis and Interaction Between Radiation Dose and Extraction Timing

Variables with statistical significance in univariable analyses were entered into the multivariable logistic regression model (Table 4). Mandibular EQD2 remained an independent predictor of ORNJ, with each 5 Gy increase corresponding to an approximately twofold increase in odds (OR 2.10; 95% CI 1.40–3.15; *p* < 0.001). The number of extracted teeth also remained independently associated with ORNJ (OR 1.16 per additional tooth; 95% CI 1.06–1.36; *p* = 0.012), whereas hemoglobin level showed a significant inverse association (OR 0.62 per g/dL increase; 95% CI 0.44–0.88; *p* = 0.007). Extraction timing after CCRT was also independently associated with ORNJ risk (OR 1.09 per month; 95% CI 1.01–1.17; *p* = 0.022).

**Figure 2:**
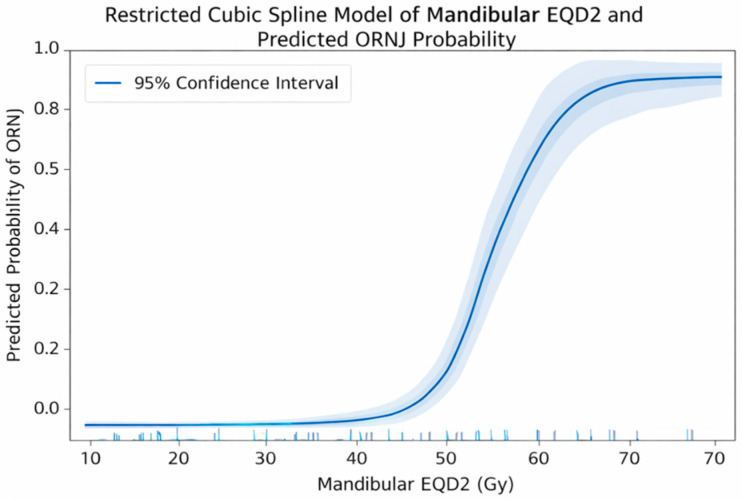
Restricted cubic spline analysis of mandibular EQD2 and predicted ORNJ probability. The spline model demonstrates a steep nonlinear increase in ORNJ risk with increasing mandibular EQD2.

Restricted cubic spline modeling demonstrated a progressive nonlinear increase in predicted ORNJ probability with increasing mandibular EQD2 (Figure 2).

**Figure 3:**
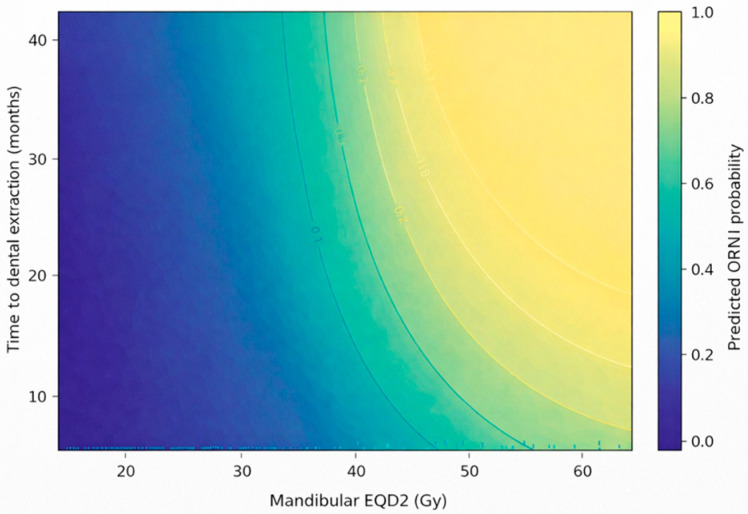
Interaction between mandibular radiation dose and dental extraction timing in predicting ORNJ. Predicted probability of ORNJ derived from logistic regression modeling, including an interaction between mandibular EQD2 and time to dental extraction. The color gradient represents increasing predicted risk. Tick marks along the lower margin indicate the distribution of individual observations.

Building on this dose–response relationship, the interaction term between mandibular EQD2 and extraction timing was statistically significant (OR = 1.07; 95% CI = 1.03–1.11; *p* = 0.023), indicating that the association between extraction timing and ORNJ varied with mandibular radiation exposure. To further characterize this interaction, predicted ORNJ probabilities were visualized across combinations of mandibular EQD2 and extraction timing (Figure 3). The interaction surface demonstrated that the influence of delayed dental extraction on ORNJ probability became more pronounced with higher levels of mandibular radiation exposure, whereas the effect of extraction timing was less pronounced at lower dose levels. This pattern was further supported by stratified analyses using ROC-derived thresholds, which demonstrated a marked increase in ORNJ rates with delayed extraction at higher mandibular dose levels (Supplementary Table S1).

### 3.6. Model Validation

Internal validation of the multivariable model was performed using bootstrap resampling with 1000 iterations, demonstrating stable model estimates without evidence of substantial overfitting. Model calibration, assessed using the Hosmer–Lemeshow goodness-of-fit test, indicated adequate agreement between predicted and observed ORNJ probabilities.

Overall, these findings indicate that ORNJ risk varies with the combined effects of mandibular radiation dose and extraction timing. This interaction-based pattern informed the subsequent interpretation of timing effects within the context of mandibular radiation exposure.

## 4. Discussion

In this retrospective cohort of 247 patients with LA-NPC treated with definitive CCRT and without pre-CCRT dental extraction, we evaluated the relationship between post-CCRT dental extraction timing and mandibular ORNJ. Multivariable analysis demonstrated that mandibular radiation dose (EQD2) emerged as an independent predictor of ORNJ. Importantly, the observed interaction suggests that although extraction timing was independently associated with ORNJ risk, this association was significantly modified by mandibular radiation dose.

An important finding of this study is that the association between post-CCRT dental extraction timing and ORNJ risk is not uniform but is significantly modified by the level of mandibular radiation exposure. Mandibular radiation dose, expressed as EQD2, was independently associated with ORNJ risk, with each 5 Gy increase corresponding to approximately a twofold increase in odds. Recent contemporary series and guideline-based reviews in the IMRT era likewise continue to identify mandibular radiation exposure as a major contributor of ORNJ risk despite advances in treatment delivery and supportive care [24]. A statistically significant interaction was observed between mandibular dose and extraction timing, indicating that the effect of extraction timing is conditional on radiation exposure. In exploratory stratified analyses, ORNJ occurred in 24.1% of patients undergoing dental extraction ≥10 months after CCRT compared with 5.2% among those treated earlier. However, stratified analyses suggested that this temporal gradient was primarily observed among patients exposed to higher mandibular radiation doses, in whom ORNJ incidence increased from 11.3% with earlier extraction to 31.1% with delayed extraction, whereas rates remained relatively low across timing categories at lower dose levels.

Within this multifactorial framework, additional systemic and local factors further modulate the risk of ORNJ. In addition to radiation dose and extraction timing, both baseline hemoglobin level and the number of extracted teeth emerged as independent predictors of ORNJ, further supporting the multifactorial nature of this complication. Lower hemoglobin levels may reduce systemic oxygen-carrying capacity, potentially exacerbating radiation-induced tissue hypoxia and impairing wound healing within the already compromised mandibular microenvironment, consistent with established radiobiological models [7,25,26]. Clinical studies have likewise identified anemia-related parameters as significant contributors to ORNJ susceptibility [27]. This observation is consistent with the central role of hypoxia in the pathophysiology of ORNJ [7,28]. Similarly, the number of extracted teeth may serve as a surrogate for the cumulative extent of local trauma and bone exposure and has been reported as an independent risk factor for ORNJ in prior clinical analyses [27]. Multiple extractions likely result in larger areas of mucosal disruption and increased demand for reparative capacity, thereby amplifying the risk of non-healing in irradiated bone [10,29]. Collectively, these findings support a multifactorial model of ORNJ susceptibility involving interactions among radiation exposure, tissue oxygenation, local traumatic burden, and host-related reparative capacity [7,25,28]. In addition, baseline oral hygiene and periodontal health may further influence post-extraction healing capacity in irradiated mandibular tissues. Emerging evidence also suggests that composite systemic inflammatory and immune-nutritional biomarkers may influence susceptibility to radiation-related tissue injury and impaired tissue healing [30,31].

Importantly, the temporal pattern observed in our cohort is highly consistent with prior literature supporting a time-dependent increase in ORNJ risk following radiotherapy. In a large cohort of LA-NPC patients, dental extraction performed beyond 8 months after CCRT was associated with a markedly higher ORNJ incidence (20.4% vs. 4.1%; *p* = 0.003) [32]. Similarly, population-based analyses have demonstrated lower ORNJ risk when extractions are performed within 6 months of radiotherapy compared with later interventions [33], while broader evidence syntheses indicate a progressive increase in ORNJ incidence over time, rising from approximately 6% within the first year to 12% beyond two years [7,34]. In parallel, systematic reviews of post-CCRT oral rehabilitation have identified a critical intermediate window between 6 and 12 months, beyond which radiation-induced hypovascularity and fibrosis become increasingly pronounced [13,35]. Taken together, the proximity of our observed ~10-month threshold to previously reported 6- and 8-month cutoffs is unlikely to be entirely incidental but is consistent with a biologically continuous risk trajectory. Within this continuum, progressive microvascular compromise and impaired tissue repair may cumulatively increase susceptibility to trauma-induced necrosis over time. Recent long-term observational data further support the dynamic nature of ORNJ risk, demonstrating continued increases in ORNJ incidence years after radiotherapy completion, even in patients treated with modern techniques [36]. Variations in reported temporal thresholds across studies likely reflect cohort-specific manifestations of an underlying shared process, supporting the concept that delayed dental extraction is a dynamic, time-dependent determinant of ORNJ risk rather than a discrete categorical exposure [26,28].

From a clinical perspective, these findings indicate that post-CCRT dental extraction timing should not be considered in isolation but interpreted in conjunction with mandibular radiation dose. A uniform temporal threshold is unlikely to be appropriate across all patients. Instead, mandibular radiation dose defines the biological context within which extraction-related trauma occurs and modulates its clinical consequences. This concept is consistent with recent multidisciplinary recommendations emphasizing individualized ORNJ risk assessment that integrates dosimetric, dental, and clinical factors [12]. At lower levels of radiation exposure, the mandibular microenvironment may retain sufficient reparative capacity to tolerate dental extraction with relatively limited risk, rendering timing less critical. In contrast, at higher radiation doses—where microvascular compromise, tissue hypoxia, and fibroatrophic remodeling are more pronounced—even modest delays may substantially increase susceptibility to ORNJ. Within this framework, extraction timing should be understood not merely as an independent determinant of risk, but as a context-dependent factor whose clinical significance may increase in settings of greater radiation-induced tissue vulnerability [7,13,16].

Beyond its implications for dental decision-making, this interaction reinforces the mandible as a clinically relevant organ at risk in radiotherapy planning. The observed increase in ORNJ susceptibility with higher mandibular radiation doses supports meticulous mandibular contouring and dose-optimization strategies. In this context, these findings further support efforts to minimize unnecessary mandibular radiation exposure without compromising tumor control.

Although the exact mechanisms remain incompletely elucidated, the observed interaction may be interpreted within a radiobiological framework of progressive tissue vulnerability. At lower radiation doses, the mandibular microenvironment may retain relatively preserved vascular integrity and reparative capacity. In contrast, higher radiation doses are associated with cumulative endothelial injury, microvascular rarefaction, tissue hypoxia, and fibroatrophic remodeling, all of which impair wound-healing capacity. Within this compromised environment, delayed dental extraction may coincide with advanced tissue deterioration, increasing susceptibility to trauma-induced necrosis. Accordingly, the effect of extraction timing reflects the interaction between the timing of trauma and the evolving severity of radiation-induced tissue injury rather than an independent temporal phenomenon [6,7,28,29].

From a methodological standpoint, this study addresses several limitations of prior investigations. The use of a homogeneous cohort, exclusion of pre-CCRT dental extraction, and continuous EQD2 modeling help mitigate confounding and improve biological interpretability. The explicit inclusion of an interaction term between mandibular radiation dose and extraction timing represents a key strength, enabling formal evaluation of effect modification. In addition, standardized longitudinal oral assessments and internal validation procedures, including bootstrap resampling and calibration analysis, support the model’s robustness [17].

Several limitations of this study should be acknowledged. First, the retrospective observational design is susceptible to selection bias, information bias, and incomplete data capture, which may limit the accuracy of exposure and outcome assessment and constrain causal interpretation. Second, the single-institution setting, while ensuring treatment uniformity, may limit the generalizability of the findings to broader patient populations and clinical practices. In particular, the homogeneous NPC-specific cohort and institutional treatment protocols may limit extrapolation of these findings to broader head and neck cancer populations managed with different radiotherapy techniques, dental practices, or supportive care pathways. Third, although key clinical and treatment-related variables were systematically evaluated, potentially relevant factors influencing tissue healing—such as comorbid conditions, oral hygiene status, periodontal health, and nutritional parameters—were not fully captured in a standardized retrospective format and may have contributed to residual confounding. In addition, the number of extracted teeth may not fully reflect the overall severity and complexity of baseline oral disease burden. Fourth, external validation of the multivariable model was not performed, and therefore the observed interaction findings and overall model performance require confirmation in larger independent cohorts to establish their reproducibility and generalizability. Finally, given the observational nature of the study, the identified associations should be interpreted with appropriate caution.

Future research should aim to validate these findings in prospective, multi-institutional cohorts with standardized dental and radiotherapy protocols to enhance generalizability and confirm the observed interaction between mandibular radiation dose and extraction timing. Incorporation of time-to-event analyses and competing risk models may further refine understanding of the temporal dynamics of ORNJ development, particularly in the context of varying follow-up durations and survivorship patterns. In addition, integration of advanced imaging-based biomarkers, dose–volume substructure analysis, and quantitative assessments of mandibular vascularity and bone quality may enable more precise characterization of individual susceptibility to radiation-induced injury. Recent radiomics- and predictive-modeling-based investigations further suggest that imaging-derived and machine-learning-integrated approaches may improve individualized ORNJ risk stratification [37]. Finally, integrating dosimetric, clinical, and biological parameters within multivariable or machine-learning-based prediction frameworks may facilitate the development of clinically applicable risk-stratification tools, ultimately supporting personalized decision-making for post-CCRT dental management.

## 5. Conclusions

Post-CCRT dental extraction timing is associated with mandibular ORNJ risk, but its clinical effect appears to be significantly modified by the level of mandibular radiation exposure in patients with LA-NPC. These findings suggest that extraction timing should be interpreted in conjunction with mandibular radiation exposure rather than as an isolated determinant of risk, with direct implications for individualized risk-adapted clinical decision-making. Mandibular radiation dose represents a clinically actionable and potentially optimizable determinant of ORNJ risk, reinforcing the mandible as a critical organ at risk and supporting careful contouring and dose optimization without compromising tumor control. Taken together, these findings indicate that mandibular radiation dose substantially influences the association between post-CCRT dental extraction timing and ORNJ risk.

## Figures and Tables

**Table 1:** Baseline clinical, lifestyle, dental, and dosimetric characteristics according to ORNJ status.

Variable	Total (n = 247)	ORNJ (n = 23)	No ORNJ (n = 224)	*p* Value
Patient characteristics				
Age, years	56 (18–78)	54 (18–76)	56 (18–78)	0.706
Sex, n (%)				
Male	163 (66.0)	14 (60.9)	149 (66.5)	0.754
Female	84 (34.0)	9 (39.1)	75 (33.5)	
ECOG				
0	101 (40.9)	9 (39.1)	92 (41.1)	0.792
1	146 (59.1)	14 (60.9)	132 (58.9)	
Hemoglobin, g/dL	12.27 (7.48–17.30)	10.46 (8.72–17.30)	12.56 (7.48–17.23)	<0.001
Diabetes, n (%)	28 (11.3)	3 (13.0)	25 (11.2)	0.567
Lifestyle factors				
Tobacco use, n (%)	134 (54.3)	15 (65.2)	119 (53.1)	0.290
Alcohol use, n (%)	41 (16.6)	5 (21.7)	36 (16.1)	0.530
Tumor features				
WHO histology				
Type 2	24 (9.7)	2 (8.7)	22 (9.8)	0.672
Type 3	223 (90.3)	21 (91.3)	202 (90.2)	
T stage				
T1–2	37 (15.0)	4 (17.4)	33 (14.7)	0.459
T3–4	210 (85.0)	19 (82.6)	191 (85.3)	
N stage				
N0–1	72 (29.1)	7 (30.4)	65 (29.0)	0.816
N2–3	175 (70.9)	16 (69.6)	159 (71.0)	
Dental variables				
Number of extracted teeth	3 (1–11)	5 (2–11)	3 (1–9)	0.004
Extraction timing, months	8 (1–48)	10 (2–35)	8 (1–48)	0.010
Dosimetric variables				
Mandibular mean dose, Gy	34.4 (15.4–67.6)	56.4 (46.5–67.6)	32.8 (15.4–64.6)	<0.001
Mandibular EQD2, Gy	27.8 (10.7–68.3)	53.1 (41.0–68.3)	26.2 (10.7–54.9)	<0.001
Follow-up characteristics				
Follow-up duration, months	74 (2–158)	73 (15–151)	76 (2–158)	0.830

**Note:** Continuous variables are presented as median (range) and categorical variables as number (percentage). Comparisons were performed using the Mann–Whitney U test for continuous variables and the χ^2^ test or Fisher’s exact test for categorical variables, as appropriate. No variable-specific missing data were present in the final analytic cohort. **Abbreviations:** ORNJ, osteoradionecrosis of the jaw; ECOG, Eastern Cooperative Oncology Group; EQD2, equivalent dose in 2 Gy fractions; WHO, World Health Organization.

**Table 2:** Exploratory ROC analysis of candidate predictors for mandibular ORNJ.

Variable	AUC	95% CI	Optimal Cutoff (Youden Index)	Sensitivity	Specificity
Mandibular EQD2 (Gy)	0.885	0.831–0.939	46.5	91.7%	89.9%
Extraction timing (months)	0.691	0.573–0.799	10	67.5%	67.1%
Number of extracted teeth	0.801	0.696–0.906	3	75.2%	72.3%
Hemoglobin (g/dL) *	0.807	0.691–0.912	11.4	73.9%	72.3%

* Lower hemoglobin levels were associated with a higher probability of ORNJ. **Note:** ROC analyses were performed to illustrate the discriminative ability of candidate predictors rather than to establish definitive clinical thresholds. Continuous variables were retained in their original continuous form in subsequent regression analyses. **Abbreviations:** ORNJ, osteoradionecrosis of the jaw; ROC, receiver operating characteristic; AUC, area under the curve; CI, confidence interval; EQD2, equivalent dose in 2 Gy fractions.

**Table 3:** Comparative ORNJ rates according to exploratory ROC-derived cutoff values.

Predictor	Group	ORNJ/Total	ORNJ Rate (%)	OR (95% CI)	*p* Value	Test
Mandibular EQD2, Gy	<46.5 ≥46.5	3/149 20/98	2.0 20.4	Reference 12.7 (4.72–26.6)	<0.001	Fisher’s exact
Extraction timing (months)	<10 ≥10	10/193 13/54	5.2 24.1	Reference 5.8 (2.4–13.9)	<0.001	χ^2^
Number of extracted teeth	<3 ≥3	15/205 8/42	7.3 19.0	Reference 2.9 (1.6–5.3)	0.027	Fisher’s exact
Hemoglobin, g/dL	>11.4 ≤11.4	4/158 19/89	2.5 21.3	Reference 10.5 (3.4–31.7)	<0.001	Fisher’s exact

**Note:** Comparisons are based on ROC-derived cutoff values (Table 2) and are presented for descriptive purposes. Continuous variables were retained in regression analyses to avoid information loss associated with dichotomization. **Abbreviations:** ORNJ, osteoradionecrosis of the jaw; OR, odds ratio; CI, confidence interval; EQD2, equivalent dose in 2 Gy fractions.

**Table 4:** Univariable and multivariable logistic regression analyses for predictors of mandibular ORNJ, including the EQD2 × extraction timing interaction.

Variable	Univariable OR (95% CI)	*p* Value	Multivariable OR (95% CI)	*p* Value
Age (years)	0.99 (0.95–1.04)	0.706	—	—
Sex	0.78 (0.31–1.99)	0.754	—	—
ECOG	0.87 (0.62–1.93)	0.812	—	—
Hemoglobin (g/dL)	0.63 (0.48–0.82)	<0.001	0.62 (0.44–0.88)	0.007
Tobacco use	1.66 (0.69–4.02)	0.290	—	—
Alcohol use	1.44 (0.46–4.53)	0.530	—	—
WHO histology	1.03 (0.72–1.39)	0.884	—	—
T stage	1.34 (1.02–1.74)	0.039	1.28 (0.96–1.63)	0.17
N stage	1.11 (0.86–1.43)	0.320	—	—
Number of extracted teeth	1.23 (1.07–1.41)	0.004	1.16 (1.06–1.36)	0.012
Extraction timing (months)	1.07 (1.02–1.12)	0.009	1.09 (1.01–1.17)	0.022
Mandibular EQD2 (per 5 Gy increase)	1.93 (1.53–2.44)	<0.001	2.10 (1.40–3.15)	<0.001
Mandibular EQD2 × extraction timing	—	—	1.07 (1.03–1.11)	0.023

**Note:** Variables with *p* < 0.05 in univariable analysis were included in the multivariable model. Mandibular mean dose was excluded to avoid collinearity with EQD2. Odds ratios for mandibular EQD2 represent the change in ORNJ odds per 5 Gy increase. The interaction term reflects effect modification between mandibular radiation dose and extraction timing. **Abbreviations:** ORNJ, osteoradionecrosis of the jaw; OR, odds ratio; CI, confidence interval; ECOG, Eastern Cooperative Oncology Group; EQD2, equivalent dose in 2 Gy fractions; WHO, World Health Organization.

## Data Availability

The datasets generated and/or analyzed during the current study are available from the corresponding author on reasonable request.

## Supplementary Materials

The following supporting information can be downloaded at: https://www.mdpi.com/article/10.3390/cancers18111756/s1, Table S1: Osteoradionecrosis of the jaw (ORNJ) rates according to mandibular radiation dose and timing of post-CCRT dental extraction.
