# Supplementary material for: Mandibular Radiation Dose Modifies the Association Between Post-Chemoradiotherapy Dental Extraction Timing and Osteoradionecrosis Risk: A Retrospective Cohort Study
Source: Cancers (Basel). 2026 May 27;18(11):1756. doi: 10.3390/cancers18111756 (PMC13255894; doi:10.3390/cancers18111756)
Supplement: Supplementary file 1 [file cancers-18-01756-s001.zip › cancers-4264984-supplementary.pdf]

Supplementary Table S1. Osteoradionecrosis of the jaw (ORNJ) rates according to mandibular radiation dose and timing of post-CCRT dental extraction.

Mandibular EQD2 (Gy)	Extraction timing after RT (months)	Total (n)	ORNJ (n)	ORNJ rate (%)
< 46.5	< 10	98	1	1.0
< 46.5	≥ 10	51	2	3.9
≥ 46.5	< 10	53	6	11.3
≥ 46.5	≥ 10	45	14	31.1

Note: Cutoff values for mandibular EQD2 (46.5 Gy) and extraction timing (10 months) were derived from exploratory receiver operating characteristic (ROC) analyses using the maximum Youden index. These categorizations are presented for descriptive purposes to illustrate the interaction pattern between radiation dose and extraction timing and should not be interpreted as definitive biological thresholds. Continuous variable modeling was used in all regression analyses to avoid information loss associated with dichotomization.
Abbreviations: EQD2, equivalent dose in 2-Gy fractions.
